# Land-Use Legacies Are Important Determinants of Lake Eutrophication in the Anthropocene

**DOI:** 10.1371/journal.pone.0015913

**Published:** 2011-01-10

**Authors:** Bronwyn E. Keatley, Elena M. Bennett, Graham K. MacDonald, Zofia E. Taranu, Irene Gregory-Eaves

**Affiliations:** 1 Department of Natural Resource Sciences and McGill School of Environment, McGill University, Montréal, Canada; 2 Department of Biology, McGill University, Montréal, Canada; Mt. Alison University, Canada

## Abstract

**Background:**

A hallmark of the latter half of the 20^th^ century is the widespread, rapid intensification of a variety of anthropogenically-driven environmental changes—a “Great Acceleration.” While there is evidence of a Great Acceleration in a variety of factors known to be linked to water quality degradation, such as conversion of land to agriculture and intensification of fertilizer use, it is not known whether there has been a similar acceleration of freshwater eutrophication.

**Methodology/Principal Findings:**

Using quantitative reconstructions of diatom-inferred total phosphorus (DI-TP) as a proxy for lake trophic state, we synthesized results from 67 paleolimnological studies from across Europe and North America to evaluate whether most lakes showed a pattern of eutrophication with time and whether this trend was accelerated after 1945 CE, indicative of a Great Acceleration. We found that European lakes have experienced widespread increases in DI-TP over the 20^th^ century and that 33% of these lakes show patterns consistent with a post-1945 CE Great Acceleration. In North America, the proportion of lakes that increased in DI-TP over time is much lower and only 9% exhibited a Great Acceleration of eutrophication.

**Conclusions/Significance:**

The longer and more widespread history of anthropogenic influence in Europe, the leading cause for the relatively pervasive freshwater eutrophication, provides an important cautionary tale; our current path of intensive agriculture around the world may lead to an acceleration of eutrophication in downstream lakes that could take centuries from which to recover.

## Introduction

Global land-use change has been a dominant driving feature of the Great Acceleration (GA) [1]. Worldwide, more land has been converted to growing crops since 1945 CE than in the previous 200 years combined [2]. Efforts to feed the world's rapidly growing population have also resulted in intensification of land use, including increased application of nitrogen (N) and phosphorus (P) fertilizers [3]. Both P and N play a key role in eutrophication, one of the most widespread water quality issues [4]. In lakes, excess P has long been recognized as a primary cause of eutrophication [5], which can result in algal blooms, taste and odour problems, and declines in economic value. Eutrophication is significantly affected by land-use practices, even across large spatial scales [6].

Agricultural land use, in particular, can lead to eutrophication. Phosphorus is added to agricultural landscapes in the form of fertilizers and manures. The fate of P applied to agricultural land varies according to soil type, the kind of fertilizer applied, weather, and other factors [7]. Most soils retain a substantial portion of applied P but experience a change-point at a certain P concentration, above which they typically release P in amounts closely related to soil P concentrations [8], [9]. After a long period of excess P application, some agricultural soils may reach P concentrations at which they begin delivering much higher P loads to nearby aquatic ecosystems [10]. In many lakes this response would appear as a GA of eutrophication.

Despite the increased rate of land conversion to agriculture, the intensification of fertilizer use, and exponential human population growth [11], there has been no inter-regional evaluation of the rate of change of freshwater eutrophication. An assessment of the onset and intensity of eutrophication has been stymied by the poor spatial and temporal coverage of monitoring data; it is uncommon to find limnological monitoring records long enough to detect the full extent of eutrophication patterns. However, over the past two decades advances in paleolimnological and statistical techniques have provided alternative tools to reconstruct the trophic history of lakes (e.g. [12]). Fossil diatoms can be quantitatively linked to lake-water total phosphorus levels through multivariate regression and calibration techniques (i.e. diatom-inferred TP transfer functions, DI-TP; [13]). Using paleolimnological techniques, eutrophication patterns have been defined in many lakes of the Northern Hemisphere [13].

**Figure 1:**
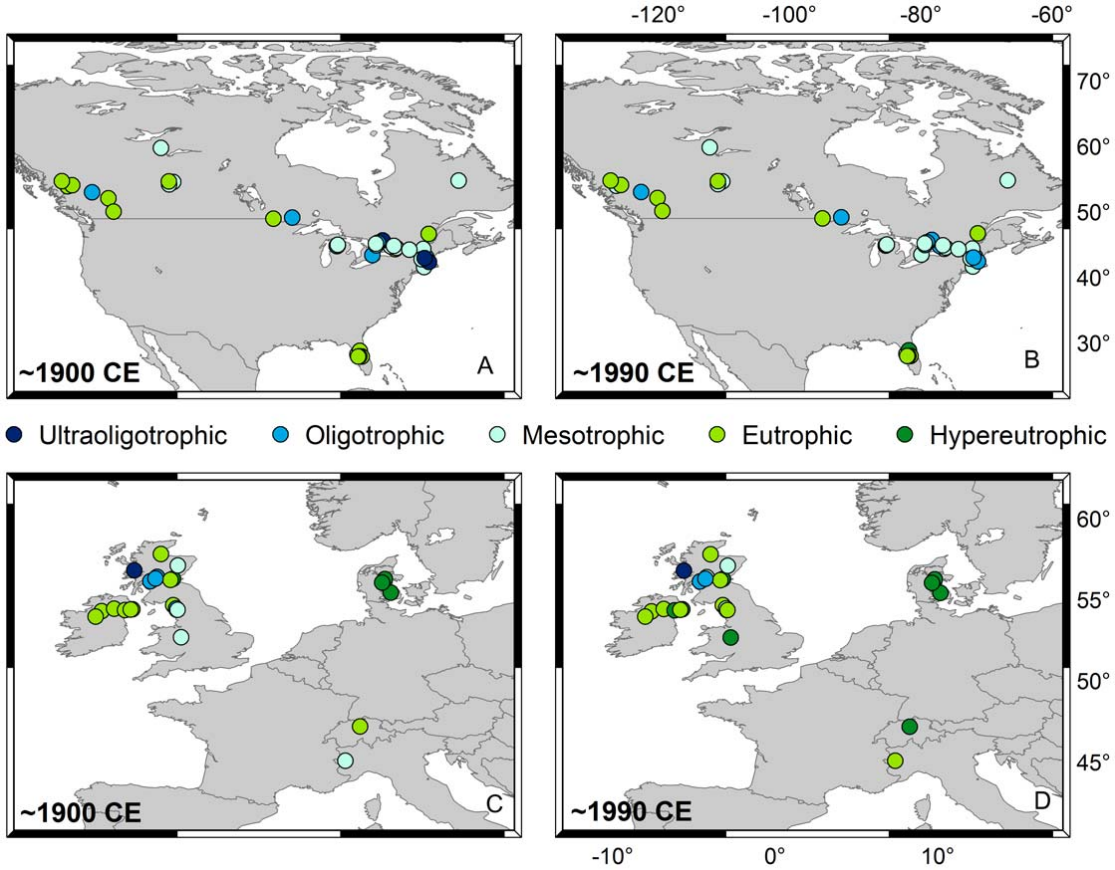
Locations and trophic status of lakes used in this study. These results are based on diatom-inferred total phosphorus estimates (DI-TP). Estimated trophic status for 43 lakes in North America ca. 1900 CE (A), and ca. 1990 CE (B) ; Estimated trophic status for 24 lakes in Europe ca. 1900 CE (C), and ca. 1990 CE (D). Lakes are classified as follows: ultraoligotrophic (dark blue); oligotrophic (medium blue); mesotrophic (light blue); eutrophic (light green); hypereutrophic (dark green).

With sufficient paleolimnological data now available from North America and Europe, we conducted a quantitative analysis of 67 existing DI-TP datasets to define the rate of change across regions and determine whether a GA of lake trophic state exists at this scale (Figure 1; Tables S1, S2). Our study had two goals: 1) to identify the proportion of lakes exhibiting a significant trend in lake trophic state over the 20^th^ century; and 2) to determine whether there is any evidence of an increase in the rate of eutrophication since ∼1945 CE, indicative of a GA. We measured trends in lake trophic state as change in DI-TP through time. We expected to find more evidence of a GA in Europe than in North America because of Europe's relatively longer history of intensive agriculture [14] as well as its higher current and historical population density [15].

## Results

Both within and between continents, we captured a broad gradient in lake and catchment morphometry, geology, climate and catchment land use, yet we detected strong differences in the mean and in the dynamics of lake trophic state between North America and Europe. Our study lakes varied in size from shallow ponds (Z_max_ = ∼1 m) to deep lakes (Z_max_ = 244 m). Lake catchments ranged from those with intensive land use (urban or agricultural) to remote environments. The lake set also spanned the trophic spectrum from ultraoligotrophic (modern TP ∼1 µg•L^−1^) to hypereutrophic (modern TP = 376 µg•L^−1^). However, we noted a striking difference between Europe and North America, where, even as early as ca. 1900, a higher proportion of European lakes were eutrophic or hypereutrophic (Figure 1).

**Figure 2:**
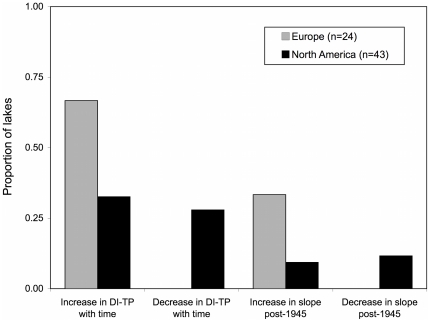
Summary results defining the trends in DI-TP through time. Linear regression and ANCOVA analyses were conducted on each time series to determine whether DI-TP showed a significant linear trend and whether there was a significant change in slope at ∼1945 CE, respectively. The proportion of all North American (black) and European sites (grey) exhibiting these patterns are summarized.

Our regression analyses revealed strong differences in the DI-TP trends between Europe and North America. Based on linear regressions, most European lakes exhibited monotonic increases in DI-TP through time (67%) and none showed a consistently declining trend in DI-TP (Figure 2, Tables S3, S4). Furthermore, we found that 33% of European lakes had a significant increase in the rate of change in DI-TP post-1945 CE and none showed a significant decrease post-1945 CE. In contrast, many North American lakes showed decreases in DI-TP over the 20^th^ century or even accelerated declines since 1945 CE (Figure 2).

**Figure 3:**
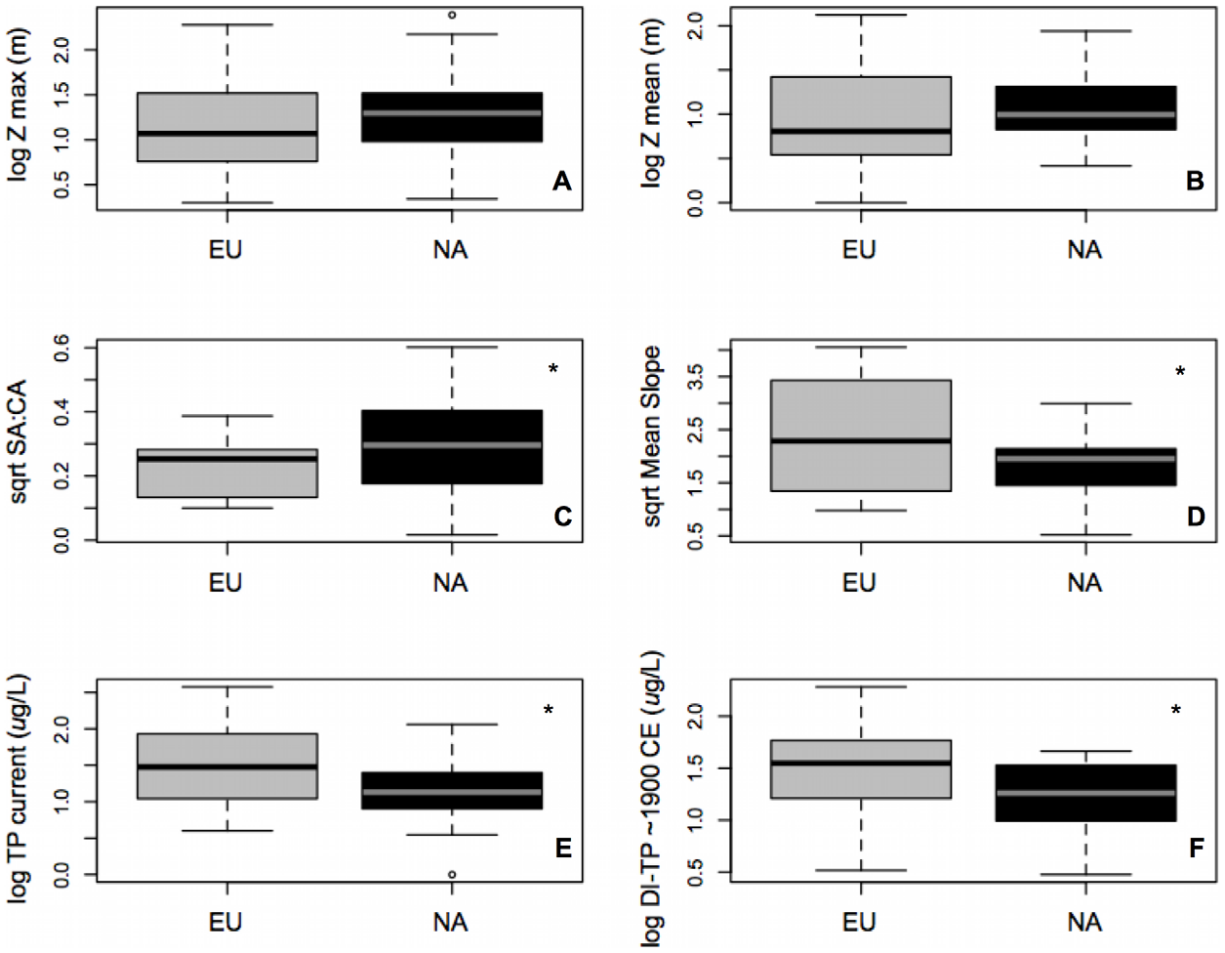
Comparison of selected limnological characteristics between Europe (grey) and North America (black). Based on t-tests, there were no significant differences between European and North American study lakes with respect to: A) maximum depth (Z_max_) and B) mean depth (Z_mean_). European lakes, however, had significantly C) lower SA:CA and D) higher mean catchment slopes. European lakes also had higher E) modern total phosphorus measurements and F) historical (ca. 1900 CE) diatom-inferred total phosphorus estimates. We also conducted t-tests on the aforementioned morphometric variables, grouping lakes according to those which showed an increase or decrease in DI-TP post-1945, but failed to detect any significant differences (data not shown).

Despite the strong differences in DI-TP trends between the two continents, North American and European lakes had many similar watershed and lake characteristics. Based on the available data, we found no significant difference in maximum or mean depth between European and North American lakes (Figure 3), nor between the North American lakes that exhibited increased or decreased DI-TP post-1945 (data not shown). However, we found that European lakes had significantly smaller surface area:catchment ratios and higher mean catchment slopes (t-tests, α<0.05; Figure 3). Both of these factors have been shown to promote nutrient transfer to downstream lakes (e.g. [16]) so these variables may have contributed to the tendency of European lakes to have significantly higher modern TP measurements and significantly higher historical DI-TP estimates than North American lakes (Figure 3).

**Figure 4:**
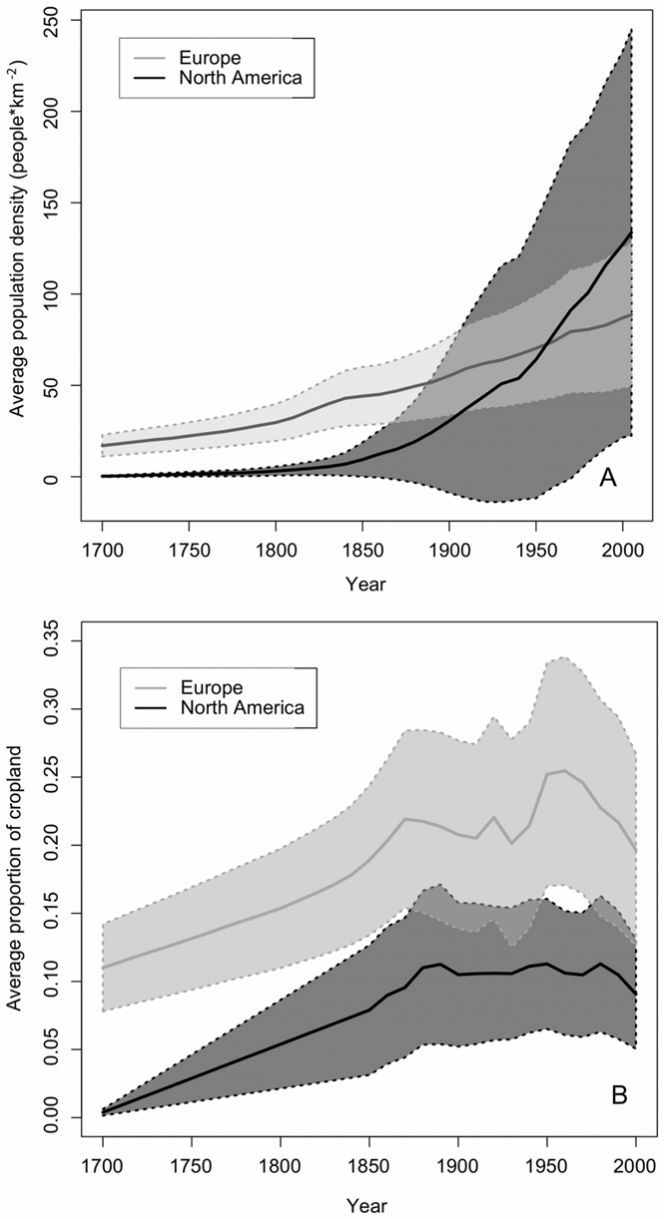
Human population densities (A) and cropland extents (B) from 1700–2000 CE. These data are shown as the average (solid lines) and 95% confidence intervals (dashed lines) of all of our North American (black) and European sites (grey).

**Figure 5:**
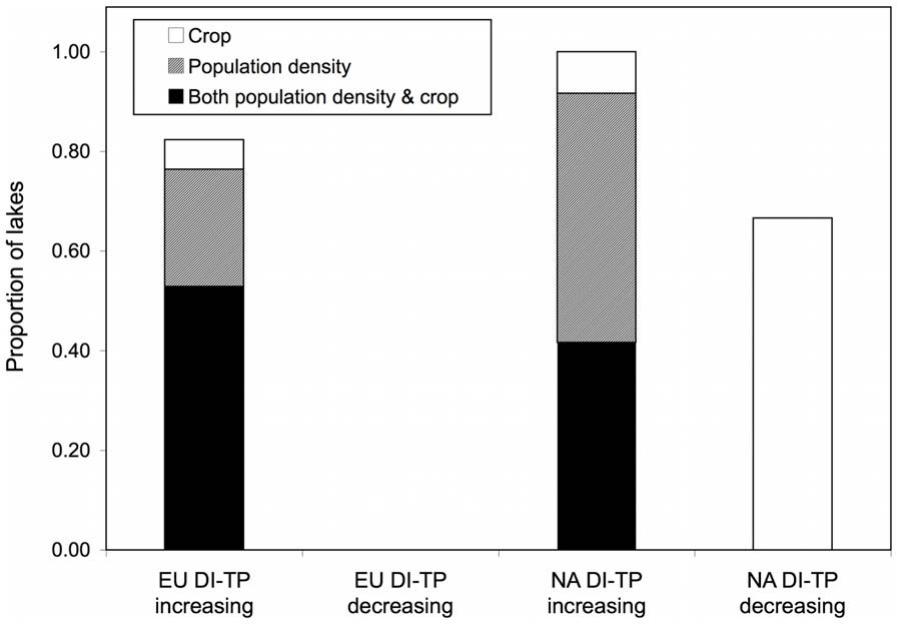
Proportion of lakes with congruent trends in population density or cropland extent and DI-TP. Lakes with congruent trends from 1900–1990 CE were defined as sites where both population density or cropland extent and DI-TP showed a significant trend over time and where these trends followed the same trajectory (i.e. same direction of change). European data are shown as EU DI-TP and North American data are shown as NA DI-TP.

Our historical analyses of population density (people·km^−2^) and the spatial extent of croplands (% of total land surface area under crops) in the areas encompassing each of our watersheds show that the North American sites had a much shorter and less intense history of landscape modification over the last three centuries than the European sites (Figure 4). In North America, we found that 100% of sites that showed an increase in DI-TP over the 20^th^ century also experienced a significant increase in human population densities and/or expansion of cropland extent whereas ∼80% of European sites showed congruent increasing trends (Figure 5). In addition, we found that ∼70% of North American lakes that decreased in DI-TP over the 20^th^ century also showed a significant decrease in cropland extent but none of the European lakes showed a decline in DI-TP.

## Discussion

Our results provide strong evidence of increasing lake trophic state in Europe over the past 100 years and some support for a GA in the rate of eutrophication within lakes in Europe during the latter half of the 20^th^ century. In contrast, we found no evidence for widespread increasing trophic status or for a GA in North America over the same period.

Given the large geographic distribution of our study sites and the similarity of key morphometric variables between continents, it is very unlikely that the differences we observed in water quality between North America and Europe are solely due to geomorphology or climate. At a landscape scale, primary determinants of lake-water phosphorus concentration are geological setting and soil type (e.g. [17]), lake and catchment morphometry (e.g. [18]), point source pollution (e.g. [19]), and predominant catchment land use, which influences non-point pollution (e.g. [20]). Given that geology and morphometry are relatively static, these factors can only explain baseline differences in lake-water nutrient concentration. Both the North American and European datasets included lakes from a variety of geomorphic characteristics (e.g. soil type, geology) and climatic zones. Therefore, as we have found for lake morphometry, geomorphology and climate are unlikely to explain continental differences between modern TP and historical DI-TP trends in Europe and North America.

The remaining factors that might drive a GA in freshwater eutrophication include anthropogenic activities such as agriculture and urbanization. Previous research shows that past periods of anthropogenic activities, especially those associated with agriculture, have greatly influenced lake trophic status (e.g. [21]). However, in most paleolimnological studies that span millennial scales, the most pronounced increases in lake-water phosphorus often occurred in the 20^th^ century (e.g. [22], [23]).

In general, Europe has greater population density as well as more pervasive landscape modifications than North America, suggesting that this may be the primary factor driving the accentuated eutrophication trends in this region. In particular, agriculture, which has had a greater extent and longer duration in Europe [14], has long been known to cause marked declines in water quality (e.g. [19], [20]). Similarly, population density, another factor known to influence P flux in freshwater systems [24], [25], has been higher in our European sites than in our North America sites for most of the past three centuries (Figure 4) [15].

While some lakes in both Europe and North America have experienced declines in surrounding cropland extents and human populations, the responsiveness of North American sites to these declines relative to European patterns are consistent with the idea that intense, long-term nutrient inputs to watershed soils can lead to over-enriched watersheds, chronic release of P, and persistent eutrophication [10]. Even after external loads of nutrients to a watershed are reduced, soils that have accumulated phosphorus can continue to act as a source of nutrients for many years. Modeling by Carpenter [10] indicated that the duration and intensity of external P load was a primary factor in determining whether a lake could recover. Our results, which show that DI-TP was more responsive to changes in watershed population density and cropland extent in North American watersheds, provide some of the first empirical support for this model.

Although we found no evidence for decreases in DI-TP in European lakes at a centennial scale, we know that many European lakes have shown declines in trophic state. Based on analyses of monitoring data from the past 5–35 years, Jeppesen et al. [26] showed that many European lakes decreased in lake-water phosphorus concentrations since the 1970s, largely due to point-source controls of nutrients. Despite nutrient controls, however, 100% of the shallow lakes and 67% of the deep lakes in Europe that were eutrophic in the ∼1970s remain eutrophic today [26]. We suggest that phosphorus that has accumulated in catchment soils from past agricultural activity continues to be a major source of nutrients to many of these lakes.

Overall, our results show that European lakes, which have had a longer history of human impact, are generally more nutrient rich than North America sites, and some European lakes show evidence of a GA in lake trophic status. Our results, together with earlier modeling research, provide an important cautionary tale for lakes in agricultural and urbanizing areas elsewhere. Specifically, we suggest that lakes situated in catchments that have had a long history of agriculture or elevated population densities may not be able to fully recover from excessive nutrient loading. Research to understand when lakes cross this ‘point of no return’ will be an important next step to managing eutrophication and restoring eutrophic lakes.

## Methods

Our study uses diatom-inferred total phosphorus data (DI-TP) that were derived as part of previous paleolimnological studies (Tables S1, S2). These data provide quantitative inferences of past trophic state dynamics from sediment records, which allows us to extend the monitoring record back through time. We searched the published literature for all available DI-TP records that fit the following criteria: 1) a quantitative DI-TP reconstruction was provided; 2) the sediment cores were independently dated using radiometric techniques; 3) the DI-TP record extended back in time until at least 1910 CE with at least three data points between ∼1900 and 1945 CE; and 4) the reconstructions were not identified by the original authors as being problematic. Studies were identified by searching ISI Web of Science, the EUROLIMPACS LAKECORES meta-database [27], and for three lakes, from colleagues' unpublished data. Although we did not intentionally restrict our search of DI-TP records to specific geographic regions, the overwhelming majority of records fitting our criteria came from North America (n = 43) and Europe (n = 24; Figure 1, Tables S1, S2). As such, we focused our paper on these two regions.

DI-TP data were most commonly provided in figure format, with DI-TP plotted either versus year or versus depth in the sediment core. Data were extracted by digitizing the graphs using the program TechDig 2.0. When DI-TP was plotted versus depth in the core rather than age, we linearly interpolated the age between dated intervals to determine age estimates for each section of the core for which a DI-TP estimate was given. In this manner, we estimated DI-TP profiles versus year for each lake.

We used spatially-explicit global datasets for historical cropland area and population density to determine whether these variables could explain trends observed in DI-TP. We used Ramankutty and Foley's [14] historical cropland area maps at 0.5 degree resolution (∼50×50 km) for the period 1700 to 2005 (updated in 2010, N. Ramankuttty, personal comm.) to estimate the spatial extent of cropland (as a percent of the total land area) in the grid cell nearest to each lake, as well as the average cropland extent for all lakes in each continent. These maps are based on historical cropland inventories obtained at the national or sub-national level, which were combined with satellite land cover data [14]. Historical population density (people·km^−2^) data for the period 1700 to 2005 were obtained from the HYDE 3.1 model by Goldewijk et al. [28] at 5-minute resolution (∼10×10 km). These population density grid cells were relatively small compared to the catchment areas of most lakes in our study and there was considerable spatial variation in population density from one cell to another in some locations. Accordingly, we estimated the local historical population density trends for each lake and continental averages for all of our lakes based on a weighted average of the grid cells adjacent to each lake. This analysis was conducted using ESRI ArcGIS v.9.3.

### Statistical analyses

We conducted linear regressions and ANCOVAs on the DI-TP data, but began our statistical analyses by assessing the normality of each DI-TP time series. Log or square-root transformations were applied to the DI-TP data where necessary (Tables S3, S4). With these data, we then ran simple linear regressions to assess whether there was a significant linear relationship between DI-TP and time over the 20^th^ century. We also conducted an ANCOVA on each DI-TP time series to determine whether there was a significant change in the slope or intercept of the regression at 1945 CE, the boundary marking the onset of the Great Acceleration for most studied variables [29]. For all linear regressions and ANCOVAs, we conducted randomization tests to quantify the significance of our results as the use of standard statistical tables may bias results in time series with significant temporal autocorrelation. We chose the “Permutation of Residuals under the Reduced Model” method as it has been shown to out-perform the raw data randomization method in terms of inflation of the type-1 error rate [30]. For further details on this method please see to Anderson and Legendre [30] and our R code (Supporting Information S1).

An important assumption of this randomization test is that the permuted residuals are independent and identically distributed. To test for independence, we conducted a Durbin-Watson test. If correlated, we pre-whitened the full model, calculated the corresponding pre-whitened model residuals, and re-evaluated the autocorrelation of residuals using the Durbin-Watson test. We then verified whether the statistical significance of the regression coefficients changed following pre-whitening, as adjusting for autocorrelation should not change the actual regression coefficients. Given that pre-whitening did not change the results of any of our autocorrelated time-series, however, we will not expand upon this further. To test for normality, we conducted Shapiro tests.

We also conducted linear regression analyses between time and the human population density or cropland time series data, applying this same randomization method. We chose not to correlate human population density or historical land cover data to DI-TP data directly because the population and land cover data alone are unlikely to accurately reflect P loading to local catchments as they do not take into account historical changes in factors such as fertilizer use, sewage treatment practices and other management changes. Instead, we focused on whether these drivers followed the same trajectory as the DI-TP data.

To determine whether there were any significant differences in morphometric or water-quality characteristics of lakes from Europe versus North America, we performed t-tests on the most-commonly reported lake characteristics (average depth (Z_mean_), maximum depth (Z_max_), surface area, surface area:catchment area ratio, mean slope of catchment, pH, modern TP, historical DI-TP ca. 1900). When estimates of surface area or catchment area were not provided, we delineated them using digital elevation models (DEM) in ArcGIS v.9.3. Catchment slope was almost never provided so we calculated average slopes for each catchment based on the DEMs.

## Supporting Information

Table S1 **Detailed information on North American lake studies used in this paper (lake name, latitude, longitude, country, province/state, and original reference).**
(XLS)

Table S2 **Detailed information on European lake studies used in this paper (lake name, latitude, longitude, country, and original reference).**
(XLS)

Table S3 **Results of linear regression and ANCOVA between time and diatom-inferred total phosphorus records (DI-TP) for North American lakes.**
(XLS)

Table S4 **Results of linear regression and ANCOVA between time and diatom-inferred total phosphorus records (DI-TP) for European lakes.**
(XLS)

Supporting Information S1 **R code for statistical analyses.**
(XLS)
